# Affibody Complex Formation: An In-Depth Thermodynamic Analysis Using Isothermal Titration Calorimetry

**DOI:** 10.3390/molecules31142500

**Published:** 2026-07-17

**Authors:** Jacek J. Walkowiak, Julian Karl

**Affiliations:** 1 DWI—Leibniz-Institute for Interactive Materials e.V., Forckenbeckstraße 50, 52074 Aachen, Germany; karl@dwi.rwth-aachen.de; 2 Institute of Technical and Macromolecular Chemistry, RWTH Aachen University, Worringerweg 2, 52074 Aachen, Germany; 3 Aachen-Maastricht Institute for Biobased Materials (AMIBM), Maastricht University, Urmonderbaan 22, 6167 RD Geleen, The Netherlands; 4 Department of Chemistry, Inorganic Chemistry III, Northern Bavarian NMR Centre, University of Bayreuth, Universitätsstrasse 30, 95440 Bayreuth, Germany

**Keywords:** protein, binding, affibody, isothermal titration calorimetry, thermodynamics

## Abstract

This study investigates the thermodynamics of binding between the affibody proteins ZTaq and anti-ZTaq across a broad temperature range, aiming to deepen the understanding of the underlying mechanisms governing their interaction. Affibodies are small, engineered proteins of notable stability and practical utility, serving as robust models for molecular recognition processes. Here, the anti-idiotypic binders ZTaq and anti-ZTaq were expressed and purified, and their interaction was characterized using isothermal titration calorimetry (ITC). The analysis revealed that the formation of the ZTaq:anti-ZTaq complex is marked by a large negative free energy of binding Δ*G*_b_ that is virtually unaffected by changes in salt concentration, in contrast to typical protein–polyelectrolyte systems where ionic strength plays a major role. Furthermore, the thermodynamic data indicated a large, negative heat capacity change Δ*C*_p_, which is primarily attributed to conformational transitions, especially the disruption of the molten-globule-like (MG) state of anti-ZTaq above 303 K. By comparing thermodynamic and structural properties with related affibody systems, the study aims to clarify how specific sequence features contribute to the exceptional binding properties of these proteins, providing new insights into protein engineering for high-affinity molecular recognition.

## 1. Introduction

Affibodies represent a distinct and versatile class of engineered binding proteins that serve as an invaluable model to understand principal protein binding interactions. In general, they are only ~6.5 kDa in size and display a three-helix bundle structure [1]. Their high expression levels in prokaryotic cells and wide range of target applications such as radio diagnostics or therapeutic use, have made them a highly researched class of molecules in protein engineering [1,2,3,4]. These affibody binders are derived from a scaffold known as the Z domain, which was itself engineered from the B domain of staphylococcal protein A (SPA), a protein known to bind and neutralize immunoglobulins [5]. The Z domain of 58 amino acids retains high thermal stability and solubility while binding to the Fc region of immunoglobulins but, unlike the parental domain, not to the Fab region [6,7]. Affibody binders are subsequently generated from a combinatorial Z-domain library in which 13 residues in the Fc-binding region on helices 1 and 2 are randomized, and targeted mutations at these positions yield individual binders with affinities comparable to antibodies and with diverse specificities, for example, toward Taq DNA polymerase, human insulin, human receptors, or even other affibody molecules [8,9,10,11]. Structural analyses of multiple affibody–target complexes demonstrate that the three-helix bundle scaffold forms a well-defined protein–protein interface in which the diversified residues on helices 1 and 2 constitute the principal contact surface, providing the structural basis for target specificity and enabling precise steric, polar, and nonpolar contacts that make affibody–target pairs an ideal model system to investigate molecular recognition and binding thermodynamics [12].

The interaction between proteins and polyelectrolytes stands as a cornerstone of contemporary molecular biophysics, with charged biopolymers, including DNA and heparin, playing a central role in numerous studies [13,14,15]. A thorough understanding of these interactions is critical, as they underpin fundamental events ranging from DNA–protein complex formation to the design of novel therapeutic agents [16,17]. Decades of research have established that electrostatic attraction, frequently manifested through the release of condensed counterions from highly charged polyelectrolytes upon protein binding, often constitutes the driving force in such systems [18,19,20]. The release of counterions generates a substantial entropic gain to the free energy of complex formation, thereby favoring the binding. Yet, the pivotal role played by water in these interactions is not so obvious at first glance, but it is just as important. Complex formation is not limited to counterion displacement but commonly involves the uptake or release of water molecules [21,22]. These subtle hydration effects can significantly influence the free energy of binding, especially at increased salt concentrations [23,24] and are intricately linked to the phenomenon of enthalpy–entropy compensation (EEC)—widely recognized as a defining characteristic of both biological and synthetic macromolecular systems [25,26,27,28]. While the thermodynamic outcomes of counterion release have been meticulously characterized, the complex interplay among salt, water activity, and binding energetics continues to be a dynamic and evolving field of research.

Recent methodological advances have shown the valuable capabilities of isothermal titration calorimetry (ITC) in the precise measurement of the binding interactions between highly charged proteins and polyelectrolytes [14,15,20,27,29]. ITC affords not only quantification of binding strength (free energy) but also serves as an indicator of underlying mechanisms—such as counterion release and associated hydration changes during complex formation [30,31].

In this study, the focus lies on the analysis of a system of anti-idiotypic binders, ZTaq and anti-ZTaq, which together provide a well-defined model for affibody–affibody recognition. Both affibodies display rapid folding kinetics and maintain remarkable temperature stability over a broad range [9]. ZTaq was originally selected to bind Taq DNA polymerase, whereas anti-ZTaq was subsequently selected to recognize ZTaq itself. In the ZTaq:anti-ZTaq complex, helices 1 and 2 of anti-ZTaq engage helices 1 and 2 of ZTaq in a perpendicular arrangement, creating an extensive interface of approximately 1.670 Å^2^. This interface is highly hydrophobic, with ~70% hydrophobic interaction content, exceeding what is typically observed in protein–protein interactions. Complex formation is accompanied by induced-fit conformational adjustments, most notably in the aromatic side chains of ZTaq. In addition to hydrophobic packing, the complex is stabilized by defined polar interactions, including hydrogen bonds involving arginine guanidinium groups, as well as close glycine–glycine contacts that may include aliphatic glycine Hα–carbonyl hydrogen bonds. The resulting complex shows moderate affinity (K_d_ ≈ 100 nM), yet the system remains a particularly clean model for dissecting how affibodies bind to each other and, by extension, for understanding general principles of affibody-mediated molecular recognition. The unbound anti-ZTaq affibody exists in a dynamic equilibrium between a molten-globule-like (MG) state and a completely unfolded state, with a melting temperature (T_m_) near 313 K [32]. Prior studies by Dincbas-Renqvist et al. have demonstrated that the temperature-dependent shift in anti-ZTaq from the MG state above 303 K has only a limited effect on the observed binding affinity in the ZTaq:anti-ZTaq complex [32]. Importantly, the stabilization of a well-ordered structure within the ZTaq:anti-ZTaq complex is associated with a pronounced loss of conformational entropy, which, in turn, opposes complex formation. In this context, the present study expands upon previous calorimetric analyses by investigating the binding interaction between the well-defined ZTaq and anti-ZTaq affibody proteins over a temperature range from 293 to 318 K, enabling a detailed thermodynamic characterization of this model system.

## 2. Results and Discussion

### 2.1. Dependence of the Binding Free Energy ΔG_b_ on Temperature and Ionic Strength

Complex formation between ZTaq and anti-ZTaq revealed a high binding constant *K*_b_, as determined by ITC. The measured heat, Δ*H*_ITC_, increases approximately linearly with increasing temperature. Table S1 (Supplementary Material) gathers all binding parameters (*N*, *K*_b_ and Δ*H*_ITC_) measured directly in the ITC experiments. As Δ*H*_ITC_ is linked with accompanying equilibria, such as the buffer ionization, it does not represent the reaction heat alone [33,34]. Therefore, its dependence on *T* and *c*_s_ will not be discussed further.

**Figure 1:**
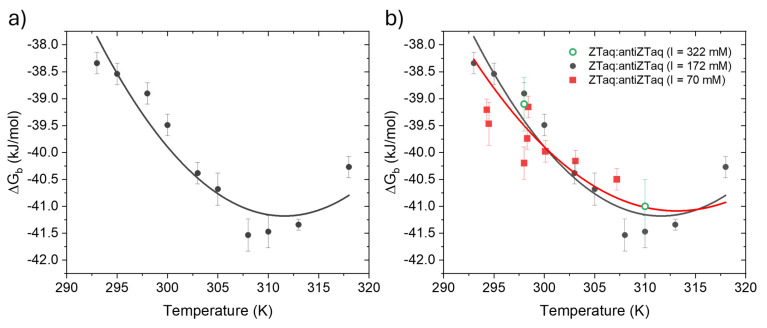
Temperature dependence of Δ*G*_b_ for the binding between ZTaq and anti-ZTaq at (**a**) I = 172 mM and (**b**) I = 70, 172, and 322 mM. Solid lines represent the fit obtained from Equation (1). The gray line represents the fit to the data points at I = 172 mM only, whereas the red line represents the fit to all data sets.

Figure 1 displays the measured binding free energy Δ*G*_b_ for ZTaq:anti-ZTaq complex formation at ionic strengths of I = 70, 172 and 322 mM, clearly showing a strong and nonlinear temperature dependence. The Δ*G*_b_ values at I = 70 mM were taken from ref. [32]. Comparison of the binding data indicates that, within the limit of error, Δ*G*_b_ is unaffected by the increase in salt concentration *c*_s_ [10]. Statistical analysis confirms this observation: the Δ*G*_b_ values at I = 70 and 172 mM, evaluated at their corresponding temperatures, did not differ significantly at the 5% level according to the Wald test. To further support this finding, additional measurements were performed at 298 and 310 K at I = 322 mM, which yielded Δ*G*_b_ values consistent with those at lower ionic strengths. This stands in a contradiction to the usually observed strong decrease in Δ*G*_b_ with increasing *c*_s_ in studies on polyelectrolyte-protein binding [15,19,27,35]. This implies that the studied interaction is not influenced by the change in ionic strength, or such influence can be disregarded. It also indicates that the counterion release term in Equation (2) can be neglected and, to a good approximation, the dependence of Δ*G*_b_ on temperature can be analyzed based on the generalized van’t Hoff expression (Equation (1)) [36]. The binding parameters resulting from the application of Equation (1) are listed in Table 1 and are characterized by a large, negative heat capacity change ${\Delta C}_{pvH}\;=\;-4.3\;\pm \;1.2\;\mathrm{kJ/mol K}$.

Large negative Δ*C*_pvH_ for the complex formation is typically associated with significant conformational changes in the protein [14,26,27,28,29,37]. The value of Δ*C*_pvH_ in the present case is clearly determined by the shift in the anti-ZTaq unfolding equilibrium, which becomes a significant effect above 303 K [32]. This indicates that above 303 K, the MG state of the protein begins to disrupt, as expected. Nevertheless, the anti-ZTaq retains its binding properties towards ZTaq even at temperatures exceeding T_m_ = 312–323 K [5,38].

**Figure 2:**
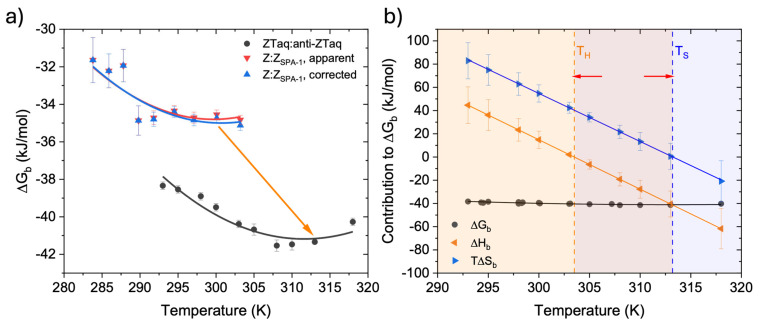
(**a**) Temperature dependence of the Δ*G*_b_ for the binding between affibodies. Solid lines represent the fit obtained from the integrated form of Equation (1). (**b**) Changes in the thermodynamic parameters (Δ*G*_b_*,* Δ*H*_b_*, T*Δ*S*_b_) that accompany the binding between ZTaq and anti-ZTaq as a function of temperature. Black dots show Δ*G*_b_. The solid black line shows the fit to Equation (1); *T*Δ*S*_b_ is shown as the blue right-pointed triangles and Δ*H*_b_ is shown as the orange left-pointed triangles. Solid blue and orange lines represent the linear fit to the data. *T*_H_ and *T*_S_ indicate the characteristic temperatures, where Δ*H*_b_ = 0 and Δ*S*_b_ = 0, respectively, dividing the graph into three regions where the binding is (from left to right) enthalpy-driven, driven by enthalpy and entropy, and driven solely by the latter factor.

Worth attention is the fact that Dincbas-Renqvist et al. in their studies on Z:Z_SPA-1_ complex formation always recorded a negative value, but at a much smaller magnitude, $\Delta C_{p}\;=\;-1.7\;\pm \;0.4\;\mathrm{kJ/mol K}$, at temperatures below 303 K [32]. However, when Equation (1) is used to fit their reported data, denoted in Table 2 as *app.* and *corr.* (see Figure 2a), a much lower value of the Δ*C*_pvH_ is obtained. Value that stands in full agreement, within the limit of error, with the results of the present analysis. This point illustrates the value of using a harmonized approach when discussing and comparing thermodynamic binding data.

By comparing the values for the characteristic temperatures *T*_H_ (above which binding is driven by entropic effects) and *T*_S_ (below which binding is driven by enthalpy) [37] a clear shift for ZTaq:anti-ZTaq, of approx. 10 degrees towards higher temperatures is visible (see Table 2). Thus, the complex formation of ZTaq:anti-ZTaq is favored by entropic effects up to 304 K. As mentioned already when introducing Equation (3), when the term related to counterion release vanishes, parameter *T*_S_ (temperature at which Δ*S*_b_ = 0) becomes the measure of hydration effects that accompany complex formation. This points out the importance and the driving role of the exchange of water molecules upon ZTaq:anti-ZTaq binding at the experimental temperature range. Finally, following Dogan et al. and comparing the percentage of unfolding of the affibodies as a function of temperature with the changes in the obtained thermodynamic parameters (Δ*G*_b_*,* Δ*H*_b_*, T*Δ*S*_b_) that accompany the ZTaq:anti-ZTaq complex formation, it becomes clear that *T_H_* represents the temperature above which unfolding starts to happen, whereas *T*_S_ marks the temperature at which the unfolding exceeds 10% [10].

### 2.2. Structural Analysis of Affibody Complexes

**Figure 3:**
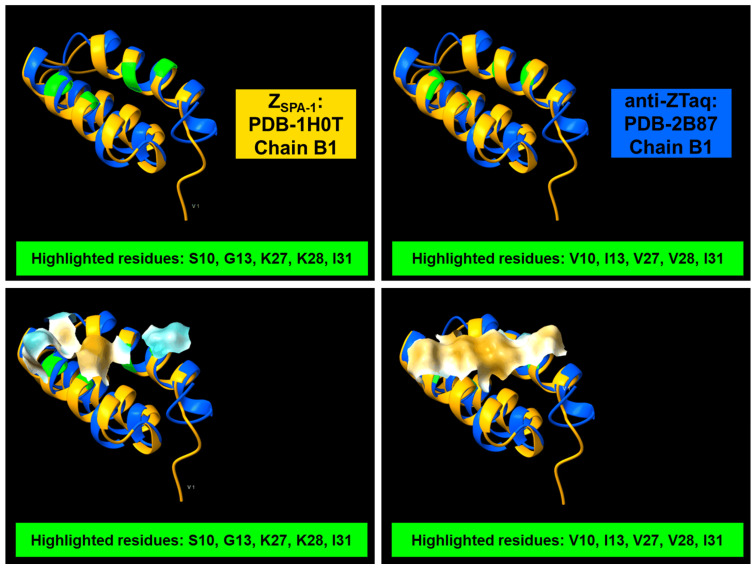
Alignment of Z_SPA-1_ (PDB:1H0T; orange) and anti-ZTaq (PDB:2B87; blue) via ChimeraX matchmaker function. Top left panel: The surface area of Z_SPA-1_ amino acid positions S10, G13, K27, K28 and I31. The positions are highlighted in green. Bottom left panel: Molecular Lipophilicity Potential (*mlp* function) of the respective amino acid positions. Top right panel: The surface area of anti-ZTaq amino acid positions V10, I13, V27, V28 and I31. The positions are highlighted in green. Bottom right panel: Molecular Lipophilicity Potential (*mlp* function) of the respective amino acid positions. The viewer’s’ perspective is centered on the binding interface area, whereby binding partners are hidden to achieve better visibility. The surface areas are shown with colors on the molecular surface, ranging from dark cyan (most hydrophilic) to white and dark gold (most lipophilic).

The cause of the improved thermal stability (with respect to *T*_H_ and *T*_S_) of the ZTaq:anti-ZTaq complex and its more negative Δ*G*_b_ may lie in specific structural differences between Z:Z_SPA-1_ and ZTaq:anti-ZTaq [5,9,39]. Detailed analysis via ChimeraX (version 1.7.1.) of ZTaq:anti-ZTaq and Z:Z_SPA-1_ complexes [32] suggests that the differences in interface complementarity can be attributed to five key amino acid substitutions in anti-ZTaq (Q10V, F13I, R27V, N28V and I31V). As shown in Figure 3, these amino acids form a large hydrophobic patch in comparison to the respective positions in Z_SPA-1_ (S10V, G13I, K27V, K28V and I31V). The analysis showed that, in Z:Z_SPA-1_, the buried surface area of hydrophobic residues (ALA, VAL, ILE, LEU, MET, PHE, TRP, PRO, and TYR) was 329.78 Å^2^, compared with 505.43 Å^2^ for hydrophilic residues (ARG, LYS, ASP, GLU, ASN, GLN, HIS, SER, and THR). Thus, the hydrophobic contribution corresponded to 65.2% of the hydrophilic one. In the ZTaq:anti-ZTaq complex, the corresponding buried surface areas were 729.94 Å^2^ and 885.79 Å^2^, respectively, so the hydrophobic contribution reached 82.4% of the hydrophilic one. Overall, the hydrophobic buried surface area was 17.2% larger in ZTaq:anti-ZTaq than in Z:Z_SPA-1_. Thus, the results of thermodynamic analysis regarding larger in magnitude Δ*G*_b_ and increased thermal stability of ZTaq:anti-ZTaq complex can be attributed to substitutions at the positions Q10V, F13I, R27V, N28V, I31V and the enlargement of the hydrophobic patch.

Compared to the earlier affibody–Protein A complex described by Wahlberg et al. [5], in which the engineered binder Z_SPA-1_ displayed only micromolar affinity and adopted an MG state that folded upon target binding, the affibody–affibody systems reported by Lendel et al. [32] achieved markedly higher affinity (with *K*_d_ of ~100 nM) while retaining stable, cooperatively folded structures in the unbound state. Structural and thermodynamic analyses further showed that these later binders rely on large, predominantly hydrophobic interfaces and enthalpy-driven interactions, with only minor entropic penalties. Together, the results illustrate a key progression: while early affibody binders demonstrated that high affinity could be generated through coupled folding on binding, subsequent designs achieved stronger binding and greater stability by preserving the folded scaffold and optimizing interface complementarity [40,41,42,43].

## 3. Materials and Methods

### 3.1. Materials

All chemicals used in this study were purchased from Carl Roth GmbH (Karlsruhe, Germany), AppliChem GmbH (Darmstadt, Germany), Sigma-Aldrich Corp. (St. Louis, MO, USA), or New England Biolabs GmbH (Frankfurt am Main, Germany), unless stated otherwise. The vector pET28a(+) was purchased from Novagen (Darmstadt, Germany) and *E. coli* BL21(DE3)-Gold competent cells were obtained from Agilent Technologies, Inc. (Santa Clara, CA, USA).

### 3.2. Production and Preparation of Anti-Idiotypic Binding Proteins ZTaq and Anti-ZTaq

ZTaq and anti-ZTaq constructs (see Table 3) were ordered and cloned into E. coli BL21 (DE3) Gold cells. The preculture (Lysogeny broth LB; 10 mL supplemented with 50 μg/mL kanamycin) was inoculated and incubated (310 K; 16 h). The working culture (Terrific broth TB; 500 mL supplemented with 50 μg/mL kanamycin) was prepared by diluting the inoculum in terrific broth (optical density (OD600) of 0.1) and cultivated until reaching OD600 of 0.6 (3 h; 310 K; with aeration at 160 RPM). Protein over-expression was induced by supplementing isopropyl β-D-1-thiogalactopyranoside (0.1 × 10^−3^ m final concentration; 293 K). After 20 h, cells were harvested by centrifugation (11,200× *g* for 30 min; 277 K). Cell pellets were suspended in Strep-tag II binding buffer (Tris-HCl; pH 8; 100 mM; 150 mM NaCl) and disrupted by sonication on ice (2:30 min; interval 30 s; 70% amplitude). Soluble proteins were collected after centrifugation (3200× *g* for 45 min; 277 K). The protein solution was filtered through a 0.45 µm cellulose-acetate filter (Cytiva, Marlborough, MA, USA) and loaded onto a pre-equilibrated (binding buffer) column (StrepTrap HP, 5 mL, Cytiva). After washing with binding buffer (10 cv), protein elution was achieved isocratically with elution buffer (Tris-HCl; pH 8; 100 mM; 150 mM NaCl; 2.5 mM desthiobiotin). Purification was monitored by an ÄKTAGo chromatography system with UV detection and UNICORN^TM^ 7.10 software (Cytiva Europe GmbH, Freiburg im Breisgau, Germany). Buffer exchange was done to PBS (pH 7.4) using a PD-10 gel-filtration column (Cytiva Europe GmbH). Protein concentration was determined with the BCA protein assay kit (Novagen, Merck KGaA, Darmstadt, Germany), and protein homogeneity was analyzed by sodium dodecyl sulfate polyacrylamide gel electrophoresis (SDS-PAGE; see Figure S1 in Supplementary Material) using a Tris-Glycine gel (4–20%; NuSep Inc., Germantown, MD, USA).

### 3.3. Isothermal Titration Calorimetry

ITC was carried out on a PEAQ-ITC instrument (Microcal, Northampton, MA, USA). All solutions were prepared in a PBS buffer. A total of 39 μL of (ZTaq-17x-TwinStrep) in buffer was titrated into the sample cell with 39 consecutive injections while stirring at 750 rpm, with a time interval of 150 s between each injection. The sample cell contained 200 μL of anti-ZTaq-17x-TwinStrep solution in the same buffer. Experiments were performed at ionic strengths of 172 and 322 mM and temperatures ranging from 293 to 318 K (see Tables S1 and S2, Supplementary Material). The relatively large TwinStrep tag versus the affibody scaffold is acknowledged as a limitation of the construct. Before each experiment (binding and dilution), all samples were degassed and equilibrated for several minutes at 1° below the experimental temperature. Prior to the analysis of the ITC data, the heat of protein dilution was subtracted from the corresponding heat of binding. All ITC data were fitted using the single set of independent binding sites (SSIS) model (see Figures S2–S4 and ITC data evaluation in Supplementary Material) [44,45]. Each experiment was repeated, and the good reproducibility of data was confirmed by additional experiments conducted at different ionic strength, stirring rates and time intervals between each injection (see Figures S3 and S4, and Table S3).

### 3.4. ChimeraX

The ZTaq:anti-ZTaq and Z:_ZSPA-1_ complexes were visualized and aligned using the Matchmaker and MLP functions, and their polar and apolar contributions were analyzed using the Measure BuriedArea tool. The PDB files 1H0T and 2B87 were used for Z:_ZSPA-1_ and ZTaq:anti-ZTaq, respectively.

### 3.5. Theory

Theoretical approaches to understanding complex formation between polyelectrolytes and proteins, as well as biocondensates of highly charged proteins, have been detailed in a number of papers that are hard to overlook [15,22,30,46]. Nevertheless, the key equations used for evaluating the binding free energy Δ*G*_b_ must be summarized. The binding constant *K*_b_, as determined directly by ITC [14,26,27,28,29,37], is related to Δ*G*_b_*(T,c*_s_*)* by $∆G_{b}(T,c_{s})={-\mathrm{RTlnK}}_{b}$, and can be evaluated in a model-free way using the generalized van’t Hoff equation:

(1) $$∆G_{b}=∆H_{\mathrm{b,ref}}-{\mathrm{T∆S}}_{\mathrm{b,ref}}+∆C_{p}\left[T-T_{\mathrm{ref}}-\mathrm{Tln}(\frac{T}{T_{\mathrm{ref}}})\right]$$

where Δ*H*_b,ref_ and Δ*S*_b,ref_ represent the enthalpy and the entropy of binding, at a reference temperature *T*_ref_ that can be chosen freely [47,48]. This expression allows analysis of Δ*G*_b_*(T)* for a given *c*_s_ but does not account for simultaneous *T* and *c*_s_ variation. To address these issues, a modified expression has been developed recently [22], where Δ*G*_b_ is described as a function of both *T* and *c*_s_:

(2) $$∆G_{b}({\mathrm{T, c}}_{s})={\mathrm{RT∆n}}_{\mathrm{ci}}{\mathrm{lnc}}_{s}+∆H_{0}-{\mathrm{T∆S}}_{0}+c_{s}\frac{{\mathrm{d∆C}}_{p}}{{\mathrm{dc}}_{s}}[T-T_{o}-\mathrm{Tln}(\frac{T}{T_{0}})]$$

The first term in Equation (2) represents counterion release, quantified by Δ*n*_ci_, the net number of ions released during binding. The last term, with characteristic temperature *T*_0_ and the specific heat capacity Δ*C*_p_ scaling with *c*_s_ via the coefficient $\frac{{\mathrm{d∆C}}_{p}}{{\mathrm{dc}}_{s}}$, denotes in turn the hydration effect on Δ*G*_b_. With a residual free energy of binding Δ*G*_res_ *=* Δ*H*_0_ *− T*_0_Δ*S*_0_, comprising all contributions to Δ*G*_b_ other than the ones embodied by counterion release and hydration effects. Importantly, Equation (2) resembles the generalized van’t Hoff expression when *T_S_*—temperature at which Δ*S*_b_ = 0—is used as the reference temperature *T*_ref_. The characteristic temperature *T*_0_ equals *T*_ref_ if the term due to counterion release is vanishing:

(3) $$∆G_{b}(T)=∆H_{b}(T_{S})+∆C_{p}[T-T_{S}-Tln(\frac{T}{T_{S}})]$$

In such cases, parameter *T*_S_ is a measure of the influence of hydration on complex formation [30].

## 4. Conclusions

The findings presented in this study reveal a nuanced thermodynamic profile for the interaction between ZTaq and anti-ZTaq affibody proteins, underlining their high affinity and robust thermal stability across a broad temperature range. The binding process, independent of ionic strength, diverges from classical protein–polyelectrolyte systems, where salt concentration influence is typically central to binding energetics. Comparative analysis with earlier affibody complexes highlights the impact of specific amino acid substitutions on enhancing interface hydrophobicity and increasing the thermal shift for enthalpy- and entropy-driven binding regimes. Together, these results support that enthalpy-driven binding predominates at lower temperatures, while entropic contributions become more relevant at elevated temperatures, reflecting a sophisticated balance of structure and solvent interactions inherent to affibody recognition mechanisms. This thermodynamic characterization advances understanding of affinity and stability optimization in engineered protein systems and provides a possible perspective for designing next-generation molecular recognition tools.

## Figures and Tables

**Table 1:** Thermodynamics of the ZTaq:anti-ZTaq interaction.

Affibody Complex	I (mM)	Δ*C*_pvH_ (kJ/mol K)	T_H_ (K)	T_S_ (K)
ZTaq:anti-ZTaq	172	−5.9 ± 1.5	305	312
	70, 172 and 322	−4.3 ± 1.2	304	313

**Table 2:** Thermodynamics of the Affibody interaction.

Affibody Complex	I (mM)	Δ*C*_pvH_ (kJ/mol K)	T_H_ (K)	T_S_ (K)
ZTaq:anti-ZTaq	70, 172 and 322	−4.3 ± 1.2	304	313
Z:Z_SPA-1, app._	20	−6.5 ± 3.4	294	300
Z:Z_SPA-1, corr._	20	−6.2 ± 3.5	295	301

**Table 3:** Amino acid sequences for ZTaq and anti-ZTaq constructs.

Construct	Amino Acid Sequence
ZTaq	VDNKFNKELGWATWEIFNLPNLNGVQVKAFIDSLRDDPSQSANLLAEAKKLNDAQAPKAEAAAKEAAAKEAAAKASAWSHPQFEKGGGSGGGSGGSAWSHPQFEK
anti-ZTaq	VDNKFNKERVIAIGEIMRLPNLNSLQVVAFINSLRDDPSQSANLLAEAKKLNDAQAPKAEAAAKEAAAKEAAAKASAWSHPQFEKGGGSGGGSGGSAWSHPQFEK

## Data Availability

The data presented in this study are available within the article and Supplementary Material.

## Supplementary Materials

The following Supplementary Material can be downloaded at: https://www.mdpi.com/article/10.3390/molecules31142500/s1, References [49,50,51] are cited in the Supplementary Materials.

## Abbreviations

The following abbreviations are used in this manuscript:

ZTaq and anti-ZTaq	Anti-idiotypic affibody binders
Z_SPA-1_	Anti-idiotypic affibody binding partner to Z-Domain
SPA	Staphylococcal protein A
MG	Molten-globule-like state of a protein
K_d_	Dissociation constant
K_b_	Binding constant
T_H_	Characteristic temperature at which binding enthalpy Δ*H*_b_ equals 0
T_S_	Characteristic temperature at which binding entropy Δ*S*_b_ equals 0
I (mM)	Ionic strength
V	Valine
I	Isoleucine
S	Serine
G	Glycine
K	Lysine

## References

[1] Zhang L., Zhang H. (2024). Recent advances of affibody molecules in biomedical applications. Bioorganic Med. Chem..

[2] Löfblom J., Feldwisch J., Tolmachev V., Carlsson J., Ståhl S., Frejd F.Y. (2010). Affibody molecules: Engineered proteins for therapeutic, diagnostic and biotechnological applications. FEBS Lett..

[3] Curry S.D., Bower B.M., Saemundsson S.A., Goodwin A.P., Cha J.N. (2025). Binding affinity and transport studies of engineered photocrosslinkable affibody-enzyme-nanoparticle constructs. Nanoscale Adv..

[4] Altai M., Leitao C.D., Rinne S.S., Vorobyeva A., Atterby C., Ståhl S., Tolmachev V., Löfblom J., Orlova A. (2018). Influence of molecular design on the targeting properties of ABD-fused mono- and Bi-valent anti-HER3 affibody therapeutic constructs. Cells.

[5] Wahlberg E., Lendel C., Helgstrand M., Allard P., Dincbas-Renqvist V., Hedqvist A., Berglund H., Nygren P.Å., Härd T. (2003). An affibody in complex with a target protein: Structure and coupled folding. Proc. Natl. Acad. Sci. USA.

[6] Nilsson B., Moks T., Jansson B., Abrahmsén L., Elmblad A., Holmgren E., Henrichson C., Jones T.A., Uhlén M. (1987). A synthetic IgG-binding domain based on staphylococcal protein A. Protein Eng. Des. Sel..

[7] Jansson B., Uhlén M., Nygren P.Å. (1998). All individual domains of staphylococcal protein A show Fab binding. FEMS Immunol. Med. Microbiol..

[8] Nord K., Gunneriusson E., Ringdahl J., Stahl S., Uhlen M., Nygren P. (1997). Coinbinatorial Libraries of an a-Helical Bacterial Receptor Doinain. Nat. Biotechnol..

[9] Lendel C., Dogan J., Härd T. (2006). Structural Basis for Molecular Recognition in an Affibody:Affibody Complex. J. Mol. Biol..

[10] Dogan J., Lendel C., Härd T. (2006). Thermodynamics of Folding and Binding in an Affibody:Affibody Complex. J. Mol. Biol..

[11] Eigenbrot C., Ultsch M., Dubnovitsky A., Abrahmsén L., Härd T. (2010). Structural basis for high-affinity HER2 receptor binding by an engineered protein. Proc. Natl. Acad. Sci. USA.

[12] Nygren P.Å. (2008). Alternative binding proteins: Affibody binding proteins developed from a small three-helix bundle scaffold. FEBS J..

[13] Record M.T., Lohman T.M., de Haseth P. (1976). Ion effects on ligand-nucleic acid interactions. J. Mol. Biol..

[14] Walkowiak J.J., Ballauff M., Zimmermann R., Freudenberg U., Werner C. (2020). Thermodynamic Analysis of the Interaction of Heparin with Lysozyme. Biomacromolecules.

[15] Malicka W., Haag R., Ballauff M. (2022). Interaction of Heparin with Proteins: Hydration Effects. J. Phys. Chem. B.

[16] Bergqvist S., Williams M.A., O’Brien R., Ladbury J.E. (2004). Heat Capacity Effects of Water Molecules and Ions at a Protein–DNA Interface. J. Mol. Biol..

[17] Privalov P.L., Dragan A.I., Crane-Robinson C. (2011). Interpreting protein/DNA interactions: Distinguishing specific from non-specific and electrostatic from non-electrostatic components. Nucleic Acids Res..

[18] Record M.T., Anderson C.F., Lohman T.M. (1978). Thermodynamic analysis of ion effects on the binding and conformational equilibria of proteins and nucleic acids: The roles of ion association or release, screening, and ion effects on water activity. Q. Rev. Biophys..

[19] Xu X., Angioletti-Uberti S., Lu Y., Dzubiella J., Ballauff M. (2019). Interaction of Proteins with Polyelectrolytes: Comparison of Theory to Experiment. Langmuir.

[20] Walkowiak J.J., Nikam R., Ballauff M. (2023). Adsorption of Mono- and Divalent Ions onto Dendritic Polyglycerol Sulfate (dPGS) as Studied Using Isothermal Titration Calorimetry. Polymers.

[21] Tanford C. (1969). Extension of the theory of linked functions to incorporate the effects of protein hydration. J. Mol. Biol..

[22] Walkowiak J.J., Ballauff M. (2021). Interaction of Polyelectrolytes with Proteins: Quantifying the Role of Water. Adv. Sci..

[23] Bergqvist S., Williams M.A., O’Brien R., Ladbury J.E. (2002). Reversal of halophilicity in a protein-DNA interaction by limited mutation strategy. Structure.

[24] Bergqvist S., Williams M.A., O’Brien R., Ladbury J.E. (2003). Halophilic adaptation of protein-DNA interactions. Biochem. Soc. Trans..

[25] Jen-Jacobson L., Engler L.E., Ames J.T., Kurpiewski M.R., Grigorescu A. (2000). Thermodynamic Parameters of Specific and Nonspecific Protein-DNA Binding. Supramol. Chem..

[26] Niedzwiecka A., Darzynkiewicz E., Stolarski R. (2004). Thermodynamics of mRNA 5‘ Cap Binding by Eukaryotic Translation Initiation Factor eIF4E †. Biochemistry.

[27] Ran Q., Xu X., Dey P., Yu S., Lu Y., Dzubiella J., Haag R., Ballauff M. (2018). Interaction of human serum albumin with dendritic polyglycerol sulfate: Rationalizing the thermodynamics of binding. J. Chem. Phys..

[28] Walkowiak J., Lu Y., Gradzielski M., Zauscher S., Ballauff M. (2020). Thermodynamic Analysis of the Uptake of a Protein in a Spherical Polyelectrolyte Brush. Macromol. Rapid Commun..

[29] Ran Q., Xu X., Dzubiella J., Haag R., Ballauff M. (2018). Thermodynamics of the Binding of Lysozyme to a Dendritic Polyelectrolyte: Electrostatics Versus Hydration. ACS Omega.

[30] Ballauff M. (2024). Driving Forces in the Formation of Biocondensates of Highly Charged Proteins: A Thermodynamic Analysis of the Binary Complex Formation. Biomolecules.

[31] Walkowiak J.J. (2026). Interaction of Lysozyme with Sulfated β-Cyclodextrin: Dissecting Salt and Hydration Contributions. Molecules.

[32] Dincbas-Renqvist V., Lendel C., Dogan J., Wahlberg E., Härd T. (2004). Thermodynamics of folding, stabilization, and binding in an engineered protein-protein complex. J. Am. Chem. Soc..

[33] Yu S., Xu X., Yigit C., van der Giet M., Zidek W., Jankowski J., Dzubiella J., Ballauff M. (2015). Interaction of human serum albumin with short polyelectrolytes: A study by calorimetry and computer simulations. Soft Matter.

[34] Baker B.M., Murphy K.P. (1996). Evaluation of linked protonation effects in protein binding reactions using isothermal titration calorimetry. Biophys. J..

[35] Xu X., Ran Q., Dey P., Nikam R., Haag R., Ballauff M., Dzubiella J. (2018). Counterion-Release Entropy Governs the Inhibition of Serum Proteins by Polyelectrolyte Drugs. Biomacromolecules.

[36] Papaneophytou C.P., Grigoroudis A.I., Mcinnes C., Kontopidis G. (2014). Quantification of the Effects of Ionic Strength, Viscosity, and Hydrophobicity on Protein−Ligand Binding Affinity. ACS Med. Chem. Lett..

[37] Niedzwiecka A., Stepinski J., Darzynkiewicz E., Sonenberg N., Stolarski R. (2002). Positive Heat Capacity Change upon Specific Binding of Translation Initiation Factor eIF4E to mRNA 5‘ Cap †. Biochemistry.

[38] Myrhammar A., Rosik D., Karlström A.E. (2020). Photocontrolled Reversible Binding between the Protein A-Derived Z Domain and Immunoglobulin G. Bioconjug. Chem..

[39] Eklund M., Axelsson L., Uhlén M., Nygren P.Å. (2002). Anti-idiotypic protein domains selected from protein A-based affibody libraries. Proteins Struct. Funct. Genet..

[40] Persson J., Puuvuori E., Zhang B., Velikyan I., Åberg O., Müller M., Nygren P.Å., Ståhl S., Korsgren O., Eriksson O. (2021). Discovery, optimization and biodistribution of an Affibody molecule for imaging of CD69. Sci. Rep..

[41] Woloschuk R.M., Reed P.M.M., Jaikaran A.S.I., Demmans K.Z., Youn J., Kanelis V., Uppalapati M., Woolley G.A. (2021). Structure-based design of a photoswitchable affibody scaffold. Protein Sci..

[42] Chen S.K., López-Tena M., Russo F., Watson E.E., Dockerill M., Cabello Garcia J., Barluenga S., Winssinger N. (2026). DNA–drug conjugates enable logic-gated drug delivery amplified by hybridization chain reactions. Nat. Biotechnol..

[43] Cheng Q., Wållberg H., Grafström J., Lu L., Thorell J.O., Hägg Olofsson M., Linder S., Johansson K., Tegnebratt T., Arnér E.S.J. (2016). Preclinical PET imaging of EGFR levels: Pairing a targeting with a non-targeting Sel-tagged Affibody-based tracer to estimate the specific uptake. EJNMMI Res..

[44] Yu S., Schuchardt M., Tölle M., Van Der Giet M., Zidek W., Dzubiella J., Ballauff M. (2017). Interaction of human serum albumin with uremic toxins: A thermodynamic study. RSC Adv..

[45] Archer W.R., Schulz M.D. (2020). Isothermal titration calorimetry: Practical approaches and current applications in soft matter. Soft Matter.

[46] Bukala J., Yavvari P., Walkowiak J.J., Ballauff M., Weinhart M. (2021). Interaction of linear polyelectrolytes with proteins: Role of specific charge–charge interaction and ionic strength. Biomolecules.

[47] Jen-Jacobson L., Engler L.E., Jacobson L.A. (2000). Structural and Thermodynamic Strategies for Site-Specific DNA Binding Proteins. Structure.

[48] Liu Y., Sturtevant J.M. (1997). Significant discrepancies between van’t Hoff and calorimetric enthalpies. III. Biophys. Chem..

[49] Turnbull W.B., Daranas A.H. (2003). On the Value of c: Can Low Affinity Systems Be Studied by Isothermal Titration Calorimetry?. J. Am. Chem. Soc..

[50] Indyk L., Fisher H.F. (1998). [17] Theoretical aspects of isothermal titration calorimetry. Methods in Enzymology.

[51] Lin L.N., Mason A.B., Woodworth R.C., Brandts J.F. (1993). Calorimetric studies of the binding of ferric ions to human serum transferrin. Biochemistry.

