# Supplementary material for: Affibody Complex Formation: An In-Depth Thermodynamic Analysis Using Isothermal Titration Calorimetry
Source: Molecules. 2026 Jul 17;31(14):2500. doi: 10.3390/molecules31142500 (PMC13414809; doi:10.3390/molecules31142500)
Supplement: Supplementary file 1 [file molecules-31-02500-s001.zip › molecules-4451104-supplementary.pdf]

SUPPRORTING INFORMATION

Affibody complex formation: an in-depth thermodynamic analysis using isothermal titration calorimetry

Jacek J. Walkowiak [1,2,3,4,*] **and Julian Karl** [1]

[1] DWI – Leibniz-Institute for Interactive Materials e.V, Forckenbeckstraße 50, 52074 Aachen, Germany.

[2] Institute of Technical and Macromolecular Chemistry, RWTH Aachen University, Worringerweg 2, 52074 Aachen, Germany.

[3] Aachen-Maastricht Institute for Biobased Materials (AMIBM), Maastricht University, Urmonderbaan 22, 6167 RD Geleen, the Netherlands.

[4] Department of Chemistry, Inorganic Chemistry III, Northern Bavarian NMR Centre, University of Bayreuth, Universitätsstrasse 30, Bayreuth, 95440 Germany.

AUTHOR INFORMATION

Authors

Jacek J. Walkowiak – jacek.walkowiak@uni-bayreuth.de; orcid.org/0000-0001-9172-5258

Julian Karl – karl@dwi.rwth-aachen.de;

Corresponding Author

Jacek J. Walkowiak - Department of Chemistry, Inorganic Chemistry III, and Northern Bavarian NMR Centre, University of Bayreuth, Universitätsstrasse 30, Bayreuth, 95440 Germany; orcid.org/0000-0001-9172-5258

E-Mail: jacek.walkowiak@uni-bayreuth.de

1. ISOTHERMAL TITRATION CALORIMETRY

1.1. Binding parameters, proteins SDS-PAGE and normalized ITC data

Table S1. Binding parameters for the ZTaq:anti-ZTaq complex formation as measured directly in the ITC experiments.

I (mM)	Temp. (K)	N	K_b x 10^{-5} (M^{-1})	ΔH_{ITC} (kJ/mol)	ΔG_b (kJ/mol)	[ZTaq] x 10^{-6} (M)	[anti-ZTaq] x 10^{-6} (M)	c*
172	293	0.8	68.5 ± 4.9	-20.8 ± 0.4	-38.3 ± 0.2	19.5	1.9	104
	295	0.8	66.7 ± 4.8	-24.2 ± 0.3	-38.5 ± 0.2	19.5	1.9	101
	298	0.6	63.3 ± 5.2	-32.6 ± 0.4	-38.8 ± 0.2	19.5	1.9	72
	300	0.8	75.2 ± 5.5	-26.5 ± 0.5	-39.5 ± 0.2	19.5	1.9	114
	303	0.7	95.2 ± 5.3	-41.4 ± 0.2	-40.4 ± 0.2	19.5	1.9	127
	305	0.7	92.6 ± 7.7	-32.8 ± 0.7	-40.7 ± 0.3	19.5	1.9	123
	308	0.7	110.6 ± 9.5	-53.5 ± 0.5	-41.5 ± 0.3	13.5	1.4	147
	310	0.7	98.4 ± 8.4	-39.3 ± 0.4	-41.5 ± 0.3	13.5	1.4	96
	313	0.9	79.4 ± 4.3	-38.9 ± 0.2	-41.3 ± 0.1	13.5	1.4	100
	318	0.7	41.2 ± 2.3	-87.1 ± 0.4	-40.3 ± 0.2	13.5	1.4	40
322	298	0.9	71.5 ± 14.2	-54.1 ± 4.1	-39.1 ± 0.6	31.5	3.1	20
	310	0.8	91.0 ± 24.5	-41.3 ± 2.7	-41.0 ± 0.5	25.5	2.5	23

**Weisman parameter,* $\boldsymbol{c = NK_b[anti-ZTaq]}$*, where N is the number of binding sites of anti-ZTaq,* K_b *is the binding constant, and [anti-ZTaq] is the concentration of anti-ZTaq, was used to judge the accuracy of the data.*[1]

Table S2. Details on the PBS buffer (pH 7.4 and I = 172 mM) composition.

Compound	Weight (g)	Molar mass (g/mol)	Molality (mol)
NaCl	8.0	58.4	0.137
KCl	0.2	74.6	0.003
Na_2HPO_4	1.4	142.0	0.010
KH_2PO_4	0.3	136.1	0.002

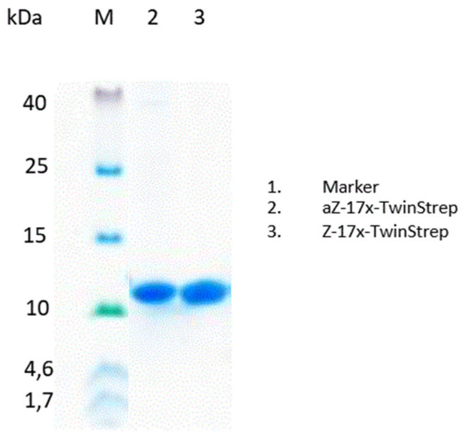

Figure S1. SDS-PAGE of ZTaq and anti-ZTaq shows pure protein fractions used for ITC experiments.

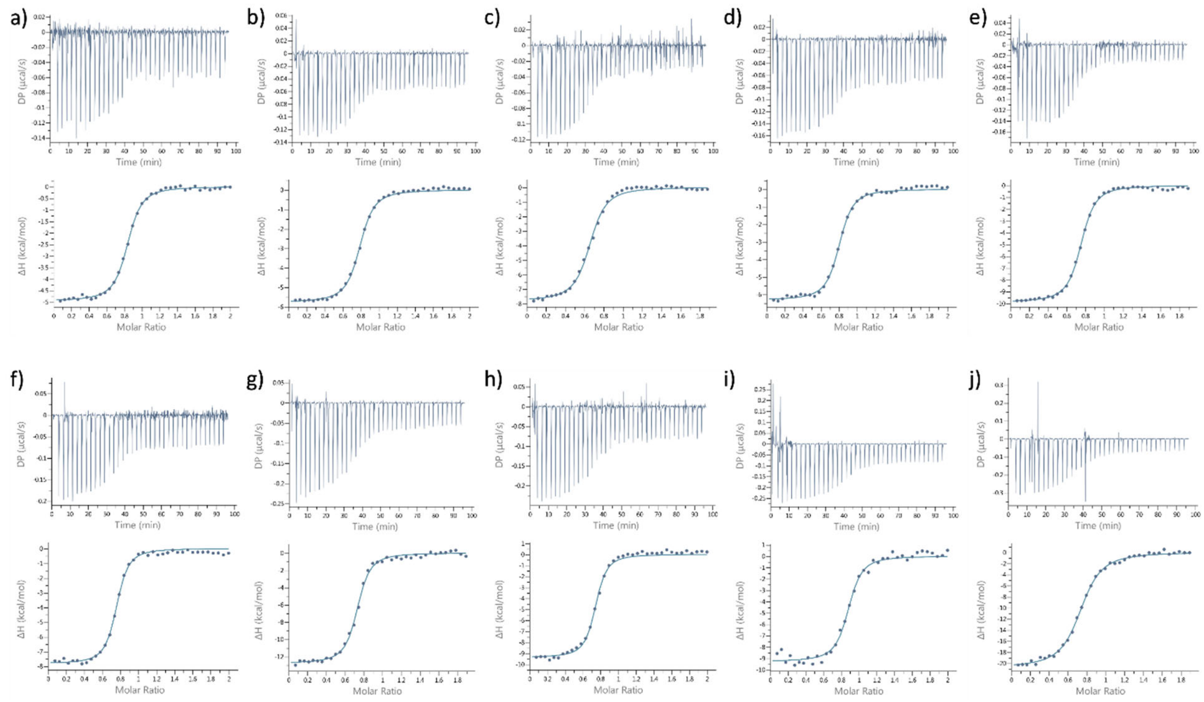

Figure S2. Normalized ITC data of the ZTaq:anti-ZTaq complex formation at (a) 293, (b) 295, (c) 298, (d) 300, (e) 303, (f) 305, (g) 308, (h) 310, (i) 313 and (j) 318 K. The bottom panels show integrated heats of adsorption with a solid blue line representing the SSIS fit. Molar Ratio always display the number of ZTaq molecules per number of anti-ZTaq molecules.

1.2. Reproducibility of the ITC data: effect of different ionic strength

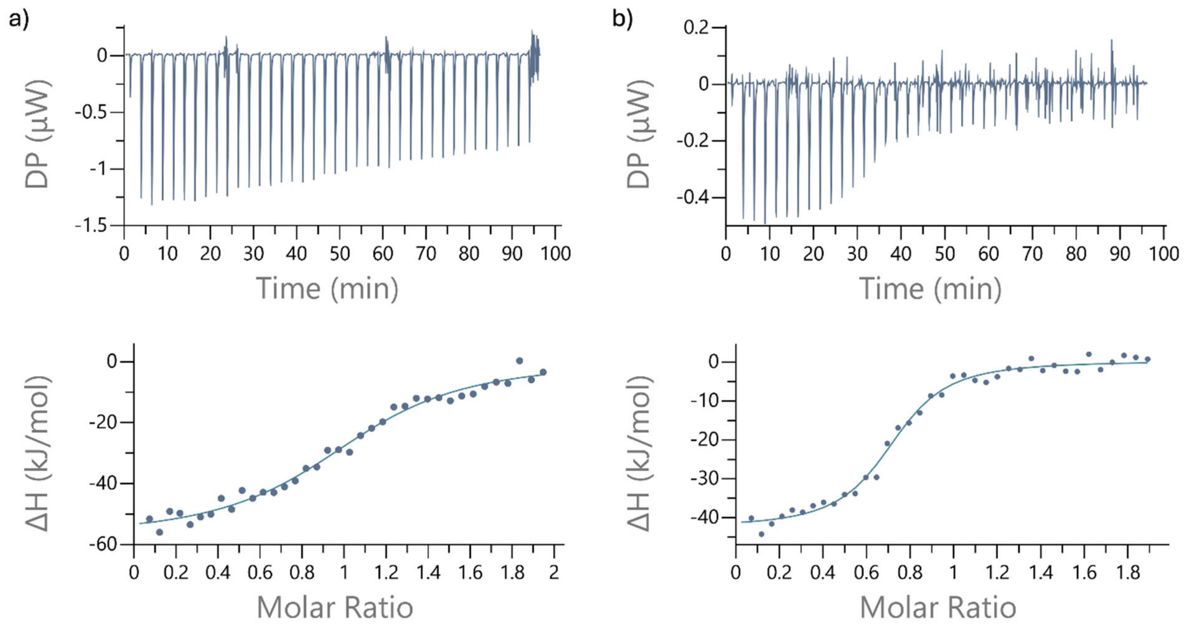

Figure S3. Normalized ITC data of the ZTaq:anti-ZTaq complex formation at I = 322 mM: (a) 298 and (b) 310 K. The bottom panels show integrated heats of adsorption with a solid blue line representing the SSIS fit. Molar Ratio always display the number of ZTaq molecules per number of anti-ZTaq molecules.

Figure S3 a show that, even at high protein concentrations (see Table S1), the heat signal for ZTaq:anti-ZTaq complex formation is significantly weakened at I = 322 mM. Although increasing the temperature can amplify the signal, as demonstrated in Figure S3 b, an ionic strength of 322 mM represents the highest value at which data can be obtained with sufficient accuracy.

1.3. Reproducibility of the ITC data: effect of different experimental conditions

<u>For standard measurements</u> performed in this study on a PEAQ-ITC instrument (Microcal, Northampton, MA): All samples were prepared in a PBS buffer. A total of 39 μL of (ZTaq-17x-TwinStrep)–buffer solution was titrated into the sample cell with 39 successive injections, with a stirring rate of 750 rpm and a time interval of 150 s between each injection. The sample cell contained 200 μL of anti-ZTaq-17x-TwinStrep solution in a matching buffer. ITC data for T = 308 K that are used for comparison are presented in Figure S3 a.

<u>For cross-reference measurements</u> performed on a PEAQ-ITC instrument (Microcal, Northampton, MA): All samples were prepared in a PBS buffer. A total of 39 μL of (ZTaq-17x-TwinStrep)–buffer solution was titrated into the sample cell with 39 successive injections, with a stirring rate of 307 rpm and a time interval of 300 s between each injection. The sample cell contained 200 μL of anti-ZTaq-17x-TwinStrep solution in a matching buffer. ITC data for T = 308 K that are used for comparison are presented in Figure S3 b.

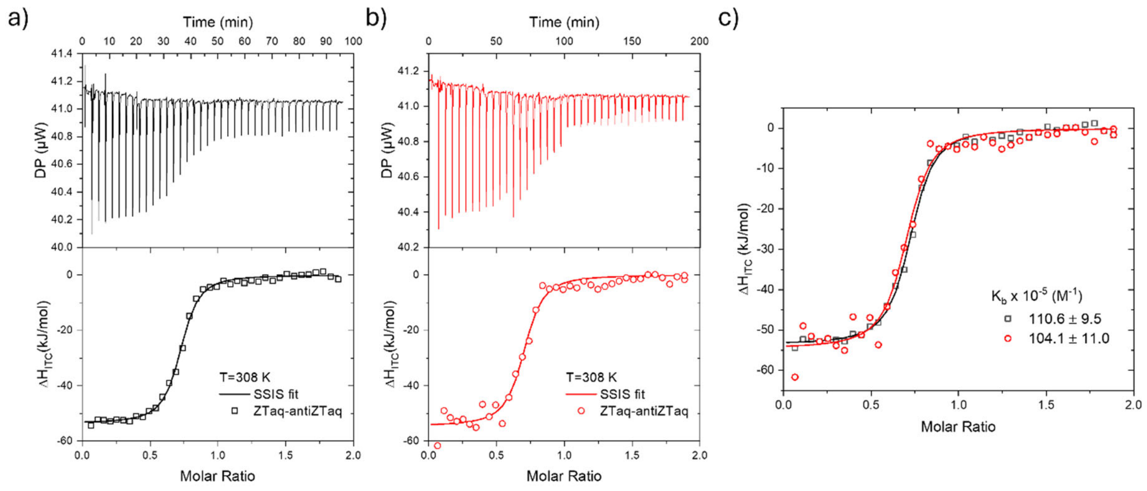

Figure S4. ITC data of the of the ZTaq:anti-ZTaq complex formation at 308 K (a) standard measurement conditions with stirring rate of 750 rpm and a time interval of 150 s between each injection, (b) cross-reference measurement conditions with stirring rate of 307 rpm and a time interval of 300 s between each injection. The bottom panels show integrated heats of adsorption with a solid line representing the SSIS fit. Molar Ratio always display the number of ZTaq molecules per number of anti-ZTaq molecules. (c) Integrated heats of the ZTaq:anti-ZTaq complex formation at 308 K. Black data points represents the binding measured at standard conditions. Red data points represent the binding measured as a cross-reference. Solid lines present the SSIS fit.

Table S3. Binding parameters (N, K_b and ΔH_{ITC}) for the ZTaq:anti-ZTaq complex formation: Effect of different experimental conditions.

I (mM)	Temp. (K)	Conditions	N	K_b x 10^{-5} (M^{-1})	ΔH_{ITC} (kJ/mol)	ΔG_b (kJ/mol)
172	308	Standard	0.8	110.6 ± 9.5	-53.5 ± 0.5	-41.5 ± 0.3
		Cross-reference	0.8	104.1 ± 11.0	-54.6 ± 1.6	-41.4 ± 0.3

Figure S4 and the binding parameters gathered in Table S3 shows that at the same condition of ionic strength and temperature the measured binding constant, K_b is not affected by the difference in the technical conditions of experiments.

2. EVALUATION OF ITC DATA

2.1 Single Set of Identical Binding Sites (SSIS) Model

The single set of independent binding site (SSIS) model is based on the Langmuir equation.[2] It assumes equilibrium between the unoccupied binding sites within the macromolecule, the number of protein molecules in solution and the macromolecule occupied binding sites. In principle it relates the fraction of adsorption sites in macromolecule containing bound protein molecules θ to the binding constant K_b:

$$\theta = \frac{K_b[P]}{1+K_b[P]} \quad \text{(S1)}$$

where [P] is the concentration of free protein molecules in solution. Since the total concentration of $[P]_t$ in the solution is known, [P] is connected to the $[P]_t$ as follows:

$$[P]_t = [P] + N\theta[M] \quad \text{(S2)}$$

For macromolecule containing N adsorption sites, θ is N_b/N where N_b represents the number of protein molecules bound per macromolecule and [M] is the total macromolecule concentration in solution. Subtracting equation (S1) into equation (S2) gives:

$$[P]_t = [P] + \frac{NK_b[P][M]}{1+K_b[P]} \quad \text{(S3)}$$

Solving of equation (S1) for [P] leads to a quadratic equation:

$$\theta^2 - \theta\left[1 + \frac{[P]_t}{N[M]} + \frac{1}{NK_b[M]}\right] = 0 \quad \text{(S4)}$$

The heat Q after each injection I is equal to:

$$Q = [M]V_0 N\theta\Delta H^{ITC} \quad \text{(S5)}$$

Solving the equation (S4) for θ and then substituting this into equation (S5) gives:

$$Q = \frac{N[M]\Delta H^{ITC}V_0}{2}\left[1 + \frac{[P]_t}{N[M]} + \frac{1}{NK_b} - \sqrt{\left(1 + \frac{[P]_t}{N[M]} + \frac{1}{NK_b[M]}\right)^2 - \frac{4[P]_t}{N[M]}}\right] \quad \text{(S6)}$$

The analysis includes the effect of the increase of the volume during titration. The experimental data are fitted by calculating the heat change of the solution ΔQ_i released with each injection I and corrected for displaced volume ΔV_i

$$\Delta Q_i = Q_i + \frac{dV_i}{V_0}\left[\frac{Q_i + Q_{i-1}}{2}\right] - Q_{i-1} \quad \text{(S7)}$$

The process of fitting experimental data involves initial guesses for N, K_b and ΔH^{ITC}; calculation of ΔQ_i for each injection and comparison of these values with the measured heat for the corresponding experimental injections; improvement in the initial values based on the Marquardt methods. The iteration of the above procedure proceeds until the satisfactory fit is achieved.[3]

REFERENCES

1. Turnbull, W.B.; Daranas, A.H. On the Value of c : Can Low Affinity Systems Be Studied by Isothermal Titration Calorimetry? *J. Am. Chem. Soc.* **2003**, *125*, 14859–14866, doi:10.1021/ja036166s.
2. Indyk, L.; Fisher, H.F. [17] Theoretical aspects of isothermal titration calorimetry. In *Methods in Enzymology*; 1998; pp. 350–364.
3. Lin, L.N.; Mason, A.B.; Woodworth, R.C.; Brandts, J.F. Calorimetric studies of the binding of ferric ions to human serum transferrin. *Biochemistry* **1993**, *32*, 9398–9406, doi:10.1021/bi00087a019.
